# Label-free and real-time monitoring of human mesenchymal stem cell differentiation in 2D and 3D cell culture systems using impedance cell sensors†

**DOI:** 10.1039/c8ra05273e

**Published:** 2018-09-04

**Authors:** Jun Ho Song, Sun-Mi Lee, Kyung-Hwa Yoo

**Affiliations:** Department of Physics, Yonsei University Seoul 03722 Republic of Korea khyoo@yonsei.ac.kr +82 2 312 7090 +82 2 2123 3887; Graduate Program for Nanomedical Science and Technology, Yonsei University Seoul 03722 Republic of Korea

## Abstract

Three dimensional (3D) stem cell culture has recently received considerable attention because it may enable the development of *in vitro* 3D tissue models. In particular, label-free and real-time monitoring of stem cell differentiation is of importance for tissue engineering applications; however, only a few non-invasive monitoring methods are available, especially for 3D cell culture. Here, we describe impedance cell sensors that allowed the monitoring of cellular behaviors in 2D and 3D cell cultures in real-time. Specifically, apparent capacitance peaks appeared in both 2D and 3D cell culture systems when human mesenchymal stem cells (hMSCs) were cultured in osteogenic induction medium. In contrast, when hMSCs were cultured in adipogenic induction medium, the capacitance increased monotonically. In addition, distinct characteristics were noted in the plots of capacitance *versus* conductance for the cells cultured in osteogenic and adipocyte induction media. These results demonstrated that the differentiation of hMSCs toward osteoblasts and adipocytes in 2D and 3D cell culture systems could be discriminated non-invasively by measuring the real-time capacitance and conductance. Furthermore, the vertical distribution of cellular activities in 3D cell cultures could be monitored in real-time using the 3D impedance cell sensors. Thus, these sensors may be suitable for monitoring the differentiation of various stem cells into different types of cells with distinct dielectric properties for tissue engineering applications.

## Abstract 2

3D impedance cell sensors are developed to monitor hMSC differentiation in label-free and real-time. Analyzing capacitance and conductance with these sensors shows that osteoblast and adipocyte lineages can be discriminated non-invasively in 3D cell culture systems.

## Graphical abstract

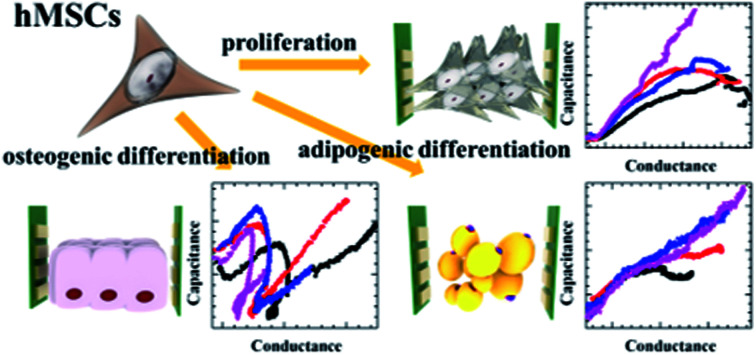

## Introduction

Three-dimensional (3D) cell cultures have been extensively investigated in the fields of drug discovery, stem cell, and tissue engineering because 3D assays simulate *in vivo* cellular conditions better than 2D cell culture systems.^1–4^ For example, traditional 2D cell monolayers are cultured on flat and rigid substrates. However, almost all cells in an *in vivo* environment are surrounded by other cells and by the extracellular matrix; therefore, 2D cell culture does not adequately mimic the natural 3D environment of cells. Accordingly, many studies have shown that cells in a 3D culture environment differ morphologically and physiologically from those in a 2D culture environment.^5–8^

Human mesenchymal stem cells (hMSCs) represent attractive candidates for use in cell therapy and regenerative medicine owing to their stable expansion and multilineage differentiation.^9–11^ When cultured in the presence of induction media that include differentiation-promoting agents and growth factors, hMSCs can differentiate into osteoblasts, adipocytes, and chondrocytes.^12,13^ Optical methods based on fluorescent probes are most often used to characterize hMSC fate, whereas a variety of biochemical assays, such as western blot, flow cytometry, and real-time polymerase chain reaction analyses are used to analyse hMSCs at the molecular level.^14–16^ However, these methods are invasive and do not provide information regarding the kinetics of cellular responses. Electric cell-substrate impedance sensors (ECIS) have thus been developed as an alternative approach to provide real-time and label-free measurements.^17–21^ With ECIS, the cells attach and spread while being cultured on the surface of the sensing electrode, and the alternating current (ac) impedance between a small sensing electrode and a large counter electrode is measured. For example, many groups have utilized ECIS to monitor various cellular activities in real-time, including adipose-derived stem cell differentiation toward osteoblasts and/or adipocytes.^22–25^ However, the electrode impedance in ECIS is influenced by the shape, adhesion, or mobility of the cells attached to the sensing electrode; therefore, this method can be applied in 2D but not in 3D cell culture systems.

**Fig. 1:**
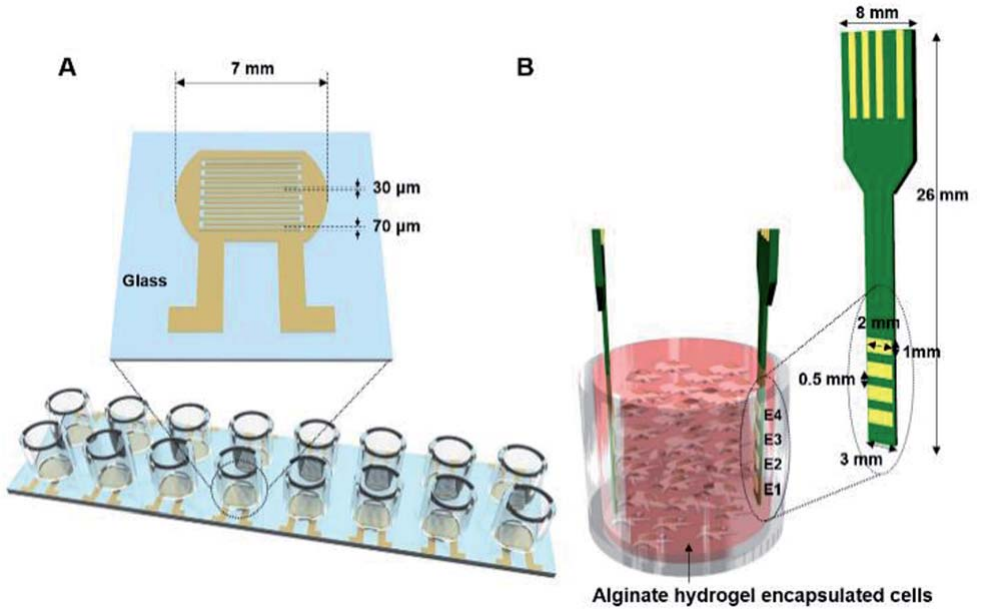
(A) A 2D impedance cell sensor array composed of 16 sensors with a pair of interdigitated electrodes and (B) a 3D impedance cell sensor consisting of four pairs of vertically aligned electrodes.

Here, we describe impedance cell sensors that can be used with both 2D as well as 3D cell culture systems. In particular, whereas a 2D impedance cell sensor has interdigitated electrodes that are fabricated on a glass substrate (Fig. 1A and S1A†), a 3D impedance cell sensor consists of four pairs of vertically aligned electrodes (Fig. 1B and S1B†). In these impedance sensors, cells are placed between two electrodes rather than on the top of the electrode; thus the dynamic information about cellular activities can be obtained by measuring changes in capacitance and conductance in real-time, where the capacitance and conductance constitute the real and imaginary portions of the impedance, respectively. We reported previously that various cellular activities, such as cell proliferation and cell death, could be monitored in real-time using these cell sensors when cells are cultured in 2D and 3D cell culture systems.^26–31^ In the present study, we further investigated whether these impedance cell sensors could discriminate between hMSCs that differentiated into osteoblasts *versus* adipocytes in real-time in both cell culture systems. Notably, the impedance cell sensors showed that osteoblast and adipocyte lineages exhibit distinct dielectric properties, allowing the label-free monitoring of hMSC differentiation in real-time using our impedance sensors.

## Materials and methods

### Cell cultures

Normal human bone marrow derived mesenchymal stem cells were purchased from Lonza (Walkersville, MD, USA), and used at passage 4 or 5 for the capacitance and conductance measurements. The hMSCs were cultured in non-induction, osteogenic induction, or adipogenic induction media. The non-induction medium consisted of Dulbecco's modified Eagle's medium (DMEM; Gibco, Rockville, MD, USA) containing 10% fetal bovine serum (PAA Laboratories, Somerset, UK), 1% penicillin–streptomycin, and 1% fungizone (Gibco). The osteogenic and adipocyte induction media were the same as non-induction medium but were supplemented with 50 μg ml^−1^l-ascorbic acid, 10 mM β-glycerophosphate, 100 nM dexamethasone (Sigma, St. Louis, MO, USA), and 500 ng ml^−1^ BMP-2 (Peprotech, Rocky Hill, NJ, USA), or with 1 μM dexamethasone, 1 μg ml^−1^ insulin, and 0.5 mM IBMX (Sigma), respectively. All cells were cultured in a humidified incubator maintained at 37 °C and 5% CO_2_.

### Alginate hydrogel scaffold

Alginate hydrogel scaffolds were prepared, as reported previously.^26^ Briefly, alginate sodium salt from brown algae (Sigma) was dissolved in DMEM culture medium to a 3% concentration (w/w) after autoclaving. For cell encapsulation, hMSCs (4 × 10^5^ cells ml^−1^) were homogeneously suspended in the 3% sodium alginate solution. The cells in the sodium alginate solution (0.8 ml) were then transferred to Millicell® cell culture inserts (diameter: 12 mm; pore size: 12 μm; Millipore, Billerica, MD USA). To crosslink the alginate hydrogel, the Millicell® inserts containing the alginate solution and the cells were immersed in 100 mM CaCl_2_-buffered MEM and incubated for 3 h in a humidified incubator maintained at 37 °C and 5% CO_2_. Before inducing cellular differentiation, the alginate hydrogel-encapsulated cells were cultured in non-induction medium (2 ml) overnight in an incubator to stabilize the cell cultures. Subsequently, the medium was changed to osteogenic or adipogenic induction medium. The media were changed every 2–3 days.

### Fabrication of 2D and 3D impedance sensors

A 2D impedance sensor array composed of 16 sensors was fabricated on a glass substrate (Fig. 1A and S1A†). Interdigitated gold electrodes with a thickness of 70 μm and a gap size of 30 μm were patterned on glass using photolithography and lift-off techniques. For cell culture, an acrylic well with a volume of 300 μl (Lab-Tek chamber slide with cover; LOT #10118584) was attached to the sensor array with polydimethylsiloxane. Prior to cell seeding, the impedance sensor array was sterilized with 70% ethanol and under ultraviolet light for 30 min.

A 3D impedance-based cell sensor consisting of four pairs of electrodes was constructed by attaching two printed circuit boards (PCBs), as reported previously.^26^ First, four parallel electrodes (1 × 2 mm^2^) with 0.5 mm spacing and four electrical connections to external connectors were patterned on a double-sided PCB and gold-plated. Next, two PCBs were placed in the impedance cell sensor vertically inside the Millicell® inserts, and a supporting stand was constructed using aluminum plates (Fig. S1B†).

### Capacitance and conductance measurements

The capacitance and conductance were measured using an LCR meter (Agilent 4294A, Santa Clara, CA, USA) with a peak-to-peak alternating current (ac) signal of 10 mV. The sensors inside the incubator and the LCR meter outside the incubator were connected *via* electrical connectors that were mounted on the side of the incubator. The capacitance and conductance were measured simultaneously for the sensors using a data acquisition/switching unit (Agilent 34970A) that was connected to the LCR meter. Data were collected every 30 min from each sensor.

### Staining with EthD-1/calcein AM

The density of live cells was estimated using a live/dead cell assay (Invitrogen, CA, USA). The cells were washed twice with phosphate buffered saline, followed by staining with 2 μM EthD-1 and 4 μM calcein AM in PBS for 30 min. Fluorescence images were obtained using a fluorescence optical microscope (IX 71, Olympus, Tokyo, Japan), and the fluorescence intensities were estimated using ImageJ software (National Institute of Health, Bethesda, MD, USA).

### Staining with ARS

To estimate the amount of calcium deposition, the cells were stained with Alizarin red S (ARS, Sigma). First, the cells in the 2D cell culture system were washed twice with PBS and fixed with 70% ethanol for 2 min. Then, the cells were stained with 2% ARS solution for 5 min, followed by three washes with deionized water. Images of hMSCs stained with ARS were examined using a fluorescence optical microscope. To quantify the amount of ARS, the stained cells were dissolved in 10% acetic acid overnight and then centrifuged at 13 000 rpm for 15 min. The supernatant was transferred to a conical tube and neutralized with 10% ammonium hydroxide. Finally, the optical density was measured at 405 nm.

To stain the cells that were cultured in the 3D cell culture system, the cells/encapsulated hydrogel was washed twice with PBS and fixed with 70% ethanol for 1 h. The cells were then stained with 2% ARS solution for 1 h, followed by three times washes with deionized water. To investigate the vertical distribution of calcium deposition, the hydrogel was sliced horizontally using a razor blade, and the sections were examined using a fluorescence optical microscope. To estimate the amount of ARS, the stained hydrogel was destained with 10% cetylpyridinium chloride monohydrate in 10 mM sodium phosphate buffer at room temperature for 15 min, and the optical density of the supernatant was measured at 570 nm.

### Staining with ORO

For Oil red O (ORO, Sigma) staining, the specimens were washed twice with PBS and fixed with 10% formaldehyde in PBS for 10 min (cells in 2D culture systems) or 30 min (cells in 3D culture systems), followed by two washes with deionized water. The hydrogel was then immersed in 60% isopropanol for 30 min (2D cultures) or 1 h (3D cultures), and stained with 0.2% ORO staining solution in 60% isopropanol for 30 min (2D cultures) or 1 h (3D cultures). The samples were rinsed three times with deionized water, and the hydrogels were sliced horizontally with a razor blade. Images of the stained sections were acquired by a fluorescence optical microscope. For quantification, the stained hydrogel was immersed in 100% isopropanol for 20 min with gentle shaking and the solution was transferred to a conical tube. The optical density was measured at 500 nm.

## Results and discussions

### Frequency dependence of the capacitance and conductance in 2D cell culture

**Fig. 2:**
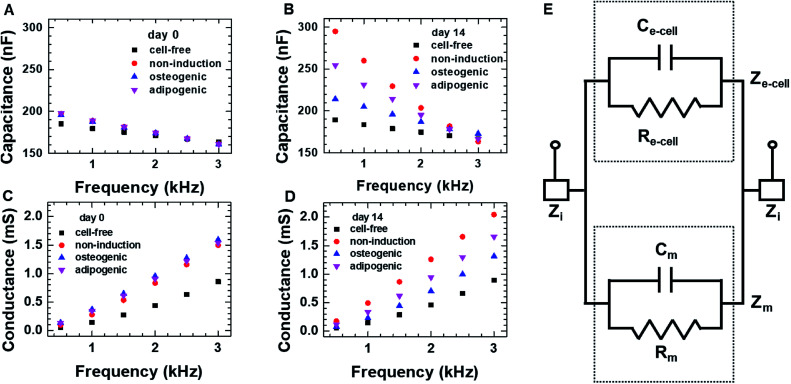
The frequency dependence of the capacitance and conductance measured on day 0 (A and C) and day 14 (B and D) for hMSCs cultured in the 2D cell culture system. In panels (A) through (D), the symbols represent the respective media used for culture; the cell-free medium was used a negative control. (E) The equivalent circuit for the 2D cell culture system; the equivalent capacitance of medium and cellular network is indicated with *C*_m_ and *C*_e-cell_, respectively, and *R*_m_ and *R*_e-cell_ are the equivalent resistance of the medium and cellular network, respectively. *Z*_i_ is the equivalent impedance of interface, and the equivalent impedance of the medium and cellular network is indicated with *Z*_m_ and *Z*_e-cell_, respectively.

We first investigated the osteogenic and adipogenic differentiation of hMSCs using 2D impedance sensor arrays (Fig. 1A and S1A†). The hMSCs (1.4 × 10^4^ cells per well) were seeded in each sensor and then cultured in non-induction, osteogenic induction, and adipogenic induction media. Fig. 2A and B show the frequency dependence of the capacitance measured for hMSCs that were cultured in various media at day 0 and 14, respectively. As the frequency increased, the capacitance decreased for all sensors, which is consistent with a dielectric relaxation theory that predicts frequency-dependent dielectric response caused by the delay in molecular polarization with respect to the ac electric field.^32,33^ However, compared to the cell-free medium (the negative control), the sensors containing hMSCs had higher capacitance values, indicating that the presence of hMSCs contributed to an increase in capacitance. At day 0, the sensors containing hMSCs exhibited similar frequency dependences regardless of the type of medium (Fig. S2†). At day 14, however, the capacitance increased differently depending on the media, particularly at low frequencies; thus, different frequency dependences were observed for different media. The frequency dependence of the conductance measured simultaneously with the capacitance at days 0 and 14 is also shown in Fig. 2C and D, respectively. The conductance showed similar overall behaviours to the capacitance, although the conductance increased with increasing frequency unlike the capacitance.

According to the dielectric relaxation theory,^32,33^ the relative complex permittivity *ε* is described by *ε* = *ε*′ − j*ε*′′, so when the applied electric field (*E*) is not very strong, the current density (*J*) is induced:

1 *J* = *σ*_S_*E* + j*ωε*_0_(*ε*′ − j*ε*′′)*E* = (*σ*_S_ + *ωε*_0_*ε*′′)*E* + j*ωε*_0_*ε*′*E*

where *σ*_S_ is the direct current (dc) conductivity of the material, *ω* is the angular frequency of the applied field, *ε*_0_ is the permittivity of free space, *ε*′ is the dielectric constant, and *ε*′′ is the loss factor of the material. Therefore, the conductivity corresponding to the real part of eqn (1) is given by *σ*_S_ + *ωε*_0_*ε*′′, whereas the capacitance is proportional to *ε*′. The *ε*′ and *ε*′′ are not independent of each other. Assuming that *σ*_S_ is very small, the *ωε*′/*ε*′′ (≈*C*/*G*) is related to 1/*τ*, where *τ*, *C*, and *G* are the characteristic relaxation time, capacitance and conductance, respectively (see ESI† for details).

The equivalent circuit for a sensor that contains cells is presented in Fig. 2E. The equivalent impedance (*Z*_e-cell_) of cells (connected in parallel and in series) between two electrodes is in parallel to the medium impedance (*Z*_m_); these together are in series with the interface impedance (*Z*_i_) at the electrode-solution interface. If *Z*_i_ is assumed to be much smaller than *Z*_e-cell_ and *Z*_m_, the total capacitance (*C*_tot_) and conductance (*G*_tot_) are approximated as *C*_tot_ ≈ *C*_m_ + *C*_e-cell_ and *G*_tot_ = 1/*R*_tot_ = 1/*R*_m_ + 1/*R*_e-cell_, respectively, where *C*_m_ and *C*_e-cell_ represent the equivalent capacitance of medium and cellular network, respectively, and *R*_m_ and *R*_e-cell_ indicate the respective equivalent resistances. It is in good agreement with the higher capacitance and conductance measured for hMSCs than for the cell-free medium.

### Real-time capacitance and conductance in 2D cell culture

**Fig. 3:**
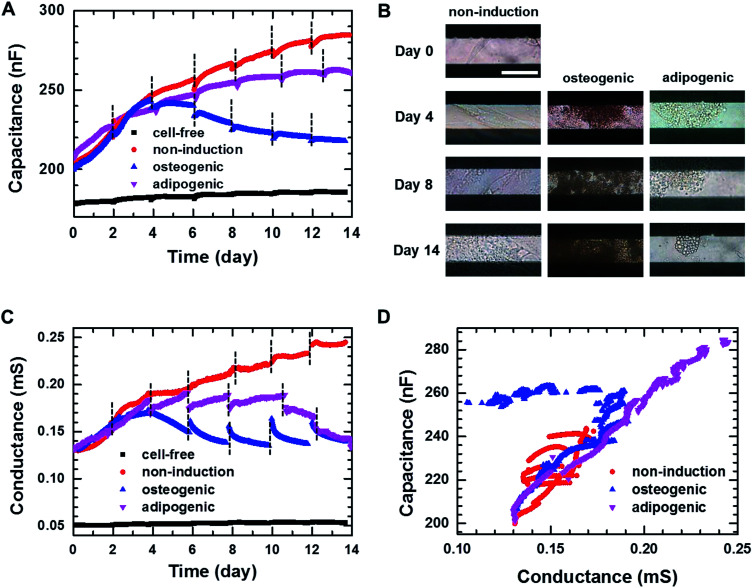
(A) Capacitance measured at *f* = 0.5 kHz over time during 2D cell cultures in the non-induction, osteogenic induction, and adipocyte induction media. The black dashed lines indicate the time point at which cell culture media were changed. (B) Time-lapse images of the hMSCs cultured in the 2D cell culture systems on the indicated days. Scale bar, 50 μm. (C) Conductance measured at *f* = 0.5 kHz over time during 2D cell cultures in the non-induction, osteogenic induction and adipocyte induction media. (D) Plot of capacitance *versus* conductance for hMSCs obtained from the hMSCs cultured in the non-induction, osteogenic induction and adipocyte induction media.

Fig. 3A shows the real-time capacitance measured at *f* = 0.5 kHz for hMSCs that were cultured in non-induction, osteogenic induction, and adipogenic induction media. The culture media were changed every 2–3 days. The capacitance similarly increased up to days 3–4 of the culture in all media, suggesting that hMSCs proliferated initially even in the induction media. After that, the capacitance of the cells in the non-induction medium increased continuously, which was ascribed to an increase in the number or volume of cells.^29^ For the cells cultured in adipogenic induction medium, the capacitance increased over 14 days similar to that observed in the non-induction medium; however, their capacitance became smaller than that of proliferated cells from days 3–4. In contrast, when cultured in the osteogenic induction medium, the cell capacitance decreased rather than increased from day 4. To investigate whether the decreased capacitance was caused by osteogenic differentiation, time-lapse optical images were acquired simultaneously with the real-time capacitance measurements (Fig. 3B). The images showed the occurrence of morphological changes in cells from day 4 in osteogenic induction medium, suggesting that the decrease in capacitance could be attributed to the onset of osteogenic differentiation.

As the capacitance in this device is closely related to the number or volume of live cells during proliferation in non-induction medium,^29^ we also estimated the number of live cells from the fluorescence images of the cells stained with ethidium homodimer-1 (EthD-1)/calcein AM at different days (Fig. S3†). The number of live cells increased from day 3 to day 7 in the osteogenic induction medium, indicating that the decreased capacitance was due to osteogenic differentiation rather than the cell death. In addition, the real-time capacitance was measured at higher frequencies than 0.5 kHz. Similar trends were observed at higher frequencies although the capacitance values were smaller (Fig. S4A†).

The real-time conductance measured at *f* = 0.5 kHz is shown in Fig. 3C. For the cells cultured in non-induction medium, the conductance exhibited similar time dependences as the capacitance. However, when the media were changed, the conductance varied considerably from day 6 for the cells in the osteogenic and adipogenic induction media, leading to apparent discontinuities. These discontinuities might be ascribed to weakened cellular adhesion on the surface caused by differentiation,^34,35^ since similar discontinuities were also reported by Bagnaninchi's group, who measured impedance using ECIS during the differentiation of adipose-derived stem cells into osteoblasts and adipocytes.^17^ In addition, the conductance for the cells in adipogenic induction medium decreased after day 4 unlike the real-time capacitance, whereas similar behaviours for these measures were observed in the osteogenic induction medium. All together these results suggested that proliferation and differentiation into osteoblasts or adipocytes could be monitored non-invasively by measuring the real-time capacitance and conductance. Furthermore, the cells with an osteoblast lineage exhibited different dielectric properties from those with an adipocyte lineage, suggesting the possibility to discriminate between osteogenic and adipogenic differentiation very early in differentiation process.

Fig. 3D shows the plot of the measured capacitance *versus* the measured conductance. A nearly linear relationship was observed in the non-induction medium, implying that *τ* maintained nearly constant value during proliferation. However, the initiation of differentiation decreased the conductance. Thus, the slope became very shallow in the adipogenic induction medium, whereas overlapping shallow slopes were observed in the osteogenic induction medium. These results suggested that *τ* of differentiated cells was longer than *τ* of proliferated cells, indicating that the impedance cell sensor allowed the discrimination between proliferation and differentiation as well as differentiation into osteoblasts *versus* adipocytes.

**Fig. 4:**
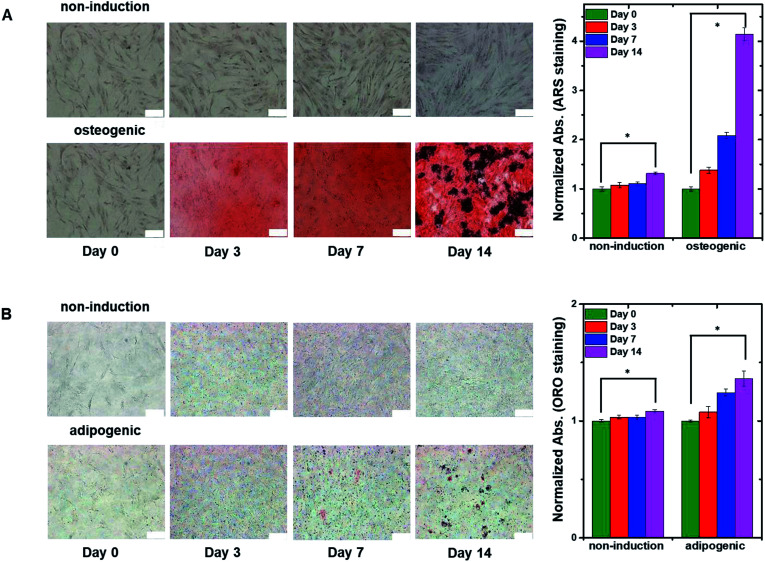
(A) Time-lapse stained images of non-induced hMSCs (top panels) and osteogenic-induced hMSCs (bottom panels) cultured in the 2D cell culture system and stained with ARS to detect calcium. The plot to the right shows normalized absorption measured on the indicated days for non-induced and osteogenic induced cells stained with ARS. (B) Time-lapse stained images of non-induced hMSCs (top panels) and adipogenic induced hMSCs (bottom panels) cultured in the 2D cell culture system and stained with ORO to detect lipids. The plot to the right shows normalized absorption measured on the indicated days for non-induced and adipogenic-induced cells stained with ORO. The data in the plots are shown as the means ± standard deviations (*n* = 5). Scale bar, 200 μm. *P < 0.001.

To confirm the differentiation of hMSCs toward osteoblasts, hMSCs were cultured in non-induction and osteogenic induction medium, and stained with Alizarin red S (ARS) at different times (Fig. 4A) to indicate calcium deposition. Positive (red) staining was nearly undetectable for 14 days in cells in the non-induction medium, whereas calcium deposition was observed on day 3 in cells in the osteogenic induction medium. These results support the idea that the decrease in capacitance was caused by osteogenic differentiation. Fig. 4B shows images of hMSCs that were cultured in non-induction and adipogenic induction medium, and stained for lipids with Oil red O (ORO) at the indicated times. Indeed, a close relationship was observed between the change in capacitance and the percentage of differentiated cells that was estimated from time-lapse stained images (Fig. S5†).

### Proliferation of hMSCs in 3D cell culture

For 3D cell culture, alginate hydrogels were utilized as a cell culture matrix. hMSCs (3 × 10^5^ cells per well) in 3% alginate solution were added into a Millicell® insert in which the 3D impedance sensor consisted of four pairs of electrodes with two parallel vertically placed electrodes (Fig. 1B). The hydrogel was crosslinked using buffered CaCl_2_ (100 mM) at 37 °C for 3 h. The buffered CaCl_2_ was then replaced with cell culture medium, and hMSCs were cultured in an incubator. Before measuring the capacitance of the sensors containing hMSCs, we measured the frequency dependence of the capacitance at different times for the cell-free alginate hydrogel as a negative control (Fig. S6A†). The frequency dependence was nearly unchanged until day 8. Thereafter, the capacitance values increased rapidly at low frequencies, unlike what was observed for the cell culture media used in 2D cell culture. To further investigate the time dependence of the capacitance for the cell-free alginate hydrogel, we measured the real-time capacitance at *f* = 0.5 kHz (Fig. S6B†). As expected, the capacitance was nearly unchanged until day 8 and then it increased rapidly, which could probably be ascribed to degradation of the alginate hydrogel.^36,37^ Hence, we analysed only data obtained before day 8 in the subsequent experiments.

**Fig. 5:**
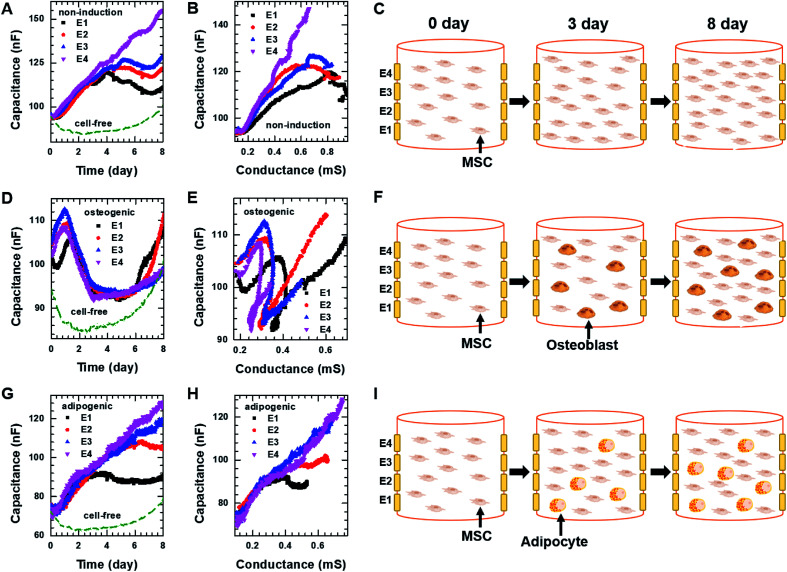
Capacitance of hMSCS measured at *f* = 0.5 kHz over time using four electrodes, E1, E2, E3, and E4 during 3D cell cultures with (A) the non-induction, (D) osteogenic induction, or (G) adipocyte induction media. Plot of capacitance *versus* conductance obtained from hMSCs in the 3D cell culture system with (B) the non-induction, (E) osteogenic induction, or (H) adipocyte induction media. Pictures illustrating what is occurring in hMSCs encapsulated in hydrogel on the indicated days during culture in the 3D cell culture system with the (C) non-induction, (F) osteogenic induction, or (I) adipocyte induction media. The real-time conductance of hMSCs in the 3D cell culture system with various media is shown in Fig. S8.†

**Fig. 6:**
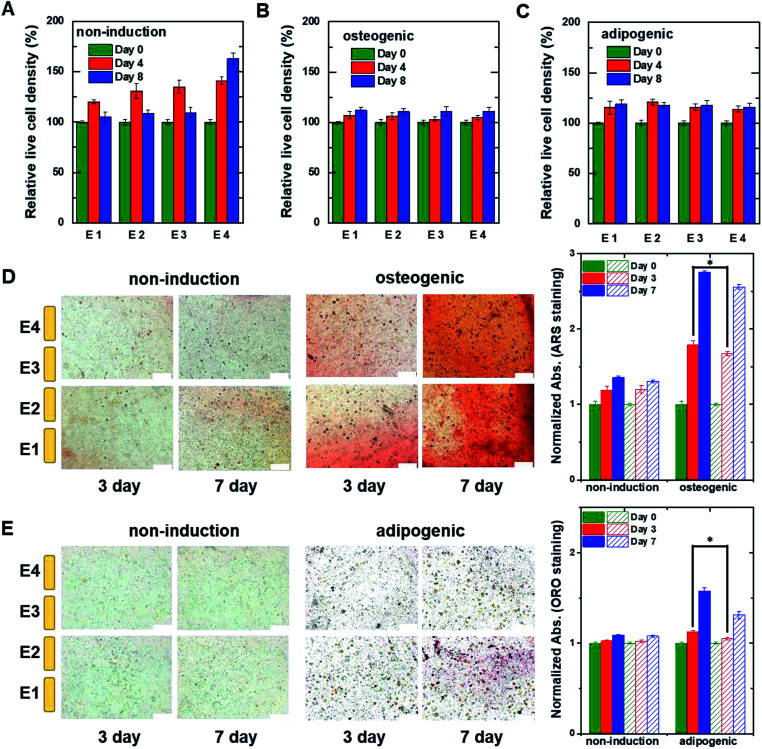
The relative live cell density of hMSCs measured at different heights within the 3D cell culture system. The hMSCs were cultured with (A) non-induction, (B) osteogenic induction, and (C) adipogenic induction media on the indicated days. The live cell densities were estimated from fluorescent images of hMSCs encapsulated in hydrogel that was sliced horizontally and stained with ethidium homodimer-1/calcein AM (Fig. S7†). The data were normalized using the optical density measured on day 0. Time-lapse stained images of hMSCs encapsulated in hydrogel that was sliced horizontally and stained with (D) ARS to detect calcium or (E) ORO to detect lipids. The hMSCs were grown in the 3D cell culture system in non-induction (top) and osteogenic induction media (bottom) for the indicated times. The plots to the right show normalized absorption for non-induced and osteogenic-induced cells stained with ARS or adipocyte-induced cells stained with ORO. The filled and dashed bars represent the data obtained from the lower region (E1 and E2) and the upper region (E3 and E4), respectively. Data are shown as the means ± standard deviations (*n* = 5). Scale bar, 200 μm. *P < 0.001.

To investigate whether the vertical distribution of cellular activities could be monitored in real-time, we measured the real-time capacitance at *f* = 0.5 kHz using four vertical electrodes (E1, E2, E3, and E4; Fig. 1B) for hMSCs cultured in the non-induction medium (Fig. 5A). The culture media were changed every 2–3 days. The capacitance measured with E4 increased continuously over time, whereas that measured with the other electrodes increased up to days 4–5, followed by a slight decrease in capacitance. To determine whether this decrease in capacitance was caused by cell death, we estimated the relative density of live cells based on the time-lapse fluorescence images of hMSCs-encapsulated in hydrogel, which was sliced horizontally and stained with EthD-1/calcein AM (Fig. 6A and S7†). For E4, the relative density of live cells increased continuously over time. However, for E1–E3, the relative density of live cells increased up to day 4 but decreased on day 8, probably because of limited culture space. These results indicated that the decrease in capacitance for E1–E3 might be due to a decrease in the number of live cells. In Fig. 5B, the capacitance is plotted as a function of the conductance (Fig. S8A†). As in 2D cell cultures, a nearly linear relationship was observed for E4. However, when the conductance was higher than about 0.6 mS, negative slopes appeared for E1–E3, potentially owing to cell death (Fig. 5C).

### Osteogenic and adipogenic differentiation of hMSCs in 3D cell culture

For osteogenic differentiation, the four vertical electrodes, E1–E4, exhibited similar capacitance peaks on days 1–1.5, as in the 2D osteogenic induction medium (Fig. 5D). However, as the relative density of live cells did not decrease before day 8 (Fig. 6B and S7†), the measurements suggested that osteogenesis in 3D cultures might be also characterized by a capacitance peak. In addition, as expected from the results of the 2D cell cultures, the plots of capacitance *versus* conductance showed shallower slopes (*i.e.*, longer *τ*) for all electrodes upon the initiation of osteogenic differentiation started (Fig. 5E). These results implied that osteogenic differentiation might be induced uniformly in the vertical direction (Fig. 5F).

In contrast, adipogenic differentiation cultures did not show capacitance peaks, and changes in the capacitance over time were electrode-dependent (Fig. 5G). The capacitance increased up to day 4 for E1 and up to day 6 for E2, then leveled off, whereas the capacitance increased continuously up to day 8 for E3 and E4. However, the number of live cells was estimated to be fairly uniform in the vertical direction (Fig. 6C and S7†). Thus, the changes over time in the capacitance for the different electrodes might be due to different degrees of adipogenic differentiation, as cultures in adipogenic induction medium had lower capacitance values than those in non-induction medium in 2D cell culture. The plots of capacitance *versus* conductance also showed a change in slope for E1 and E2, whereas a nearly linear dependence was observed for E3 and E4 (Fig. 5H), suggesting that in the lower region of the device, a certain number of hMSCs might have differentiated toward adipocytes within 8 days, whereas in the upper region, a smaller number of hMSCs might have differentiated within this period (Fig. 5I). These results demonstrate that as for 2D cell cultures, measuring the real-time capacitance and conductance might be used to discriminate between osteogenic and adipogenic differentiation in 3D cell cultures, and that the vertical distribution of cellular activities might be monitored in real-time using the impedance cell-sensors.

Fig. 6D and E show time-lapse images of hMSCs-encapsulated in hydrogel that was horizontally sliced and stained with ARS and ORO, respectively. In the case of osteogenesis, similar amounts of calcium deposition were observed in both the lower and upper regions on day 3. Conversely, in the case of adipogenesis, more lipids (stained with ORO) were observed in the lower region than in the upper one on days 3 and 7. These results are consistent with the real-time capacitance data and verify that osteogenesis was induced uniformly in the vertical direction, whereas adipogenesis was induced earlier in the lower region than in the upper region.

To investigate the repeatability of these findings, we fabricated more than five 2D impedance cell sensor arrays and 3D impedance cell sensors. Although each sensor exhibited slightly different initial capacitance and conductance values in the cell-free medium, similar results were obtained following normalization using the initial values, indicating that our impedance sensors provided reasonable repeatability (Fig. S9†). However, compared to the 2D impedance cell sensors, the 3D cell sensors yielded larger variations across the four vertical electrodes, likely because the vertical distribution of hydrogel-encapsulated cells might not be completely homogenous (Fig. S10 and S11†).

## Conclusions

In summary, we have developed 2D and 3D impedance cell sensors to monitor hMSC differentiation in real-time. These sensors showed that osteoblast and adipocyte lineages exhibit different dielectric properties that allowed us to non-invasively discriminate hMSC differentiation toward osteoblasts *versus* adipocytes in both 2D and 3D cell culture systems. Furthermore, the 3D impedance cell sensors provided dynamic information regarding cellular activities in the vertical direction. This dielectric method may therefore be suitable for label-free and real-time monitoring the differentiation of various stem cells into different types of cells with distinct dielectric properties.

## Conflicts of interest

There are no conflicts to declare.

## Supplementary Material

RA-008-C8RA05273E-s001
