# Supplementary material for: Label-free and real-time monitoring of human mesenchymal stem cell differentiation in 2D and 3D cell culture systems using impedance cell sensors
Source: RSC Adv. 2018 Sep 4;8(54):31246–54. doi: 10.1039/c8ra05273e (PMC9085567; doi:10.1039/c8ra05273e)
Supplement: RA-008-C8RA05273E-s001 [file RA-008-C8RA05273E-s001.pdf]

Electronic Supplementary Material (ESI) for RSC Advances.
This journal is © The Royal Society of Chemistry 2018

Supplementary Information

Label-free and real-time monitoring of human mesenchymal stem cell differentiation in 2D and 3D cell culture systems using impedance cell sensors

Jun Ho Song[a], Sun-Mi Lee[b], and Kyung-Hwa Yoo[ab]*

a. Department of Physics, Yonsei University, Seoul, 03722, Republic of Korea

b. Graduate Program for Nanomedical Science and Technology, Yonsei University, Seoul, 03722, Republics of Korea

Dielectric relaxation theory

According to the dielectric relaxation theory,[1] dielectric properties of a material basically reflect the electric charge movement inside the material in response to an external electric field. Assuming an applied electric field $\boldsymbol{E}$, a current density $\boldsymbol{J}$ inside the material is induced:

$$\boldsymbol{J} = \sigma_S \boldsymbol{E} + j\omega\varepsilon_0(\varepsilon' - j\varepsilon'')\boldsymbol{E} = (\sigma_S + \omega\varepsilon_0\varepsilon'')\mathbf{E} + j\omega\varepsilon_0\varepsilon'\boldsymbol{E} \qquad (1)$$

where σ_S is the direct current (dc) conductivity of the material, ω is the angular frequency of the applied field, ε_0 is the permittivity of free space, ε' is the dielectric constant, and ε'' is the loss factor of the material. For biological materials, ε usually depends on frequency. The conductivity of the material is:

$$\sigma = \sigma_S + \omega\varepsilon_0\varepsilon'' \qquad (2)$$

Dielectric relaxation is always a frequency-dependent process. A single-time-constant response is described by the Debye equation:[2]

$$\varepsilon = \varepsilon_\infty + (\varepsilon_S - \varepsilon_\infty)/(1 + j\omega\tau) \qquad (3)$$

where ε_∞ is the dielectric constant at frequencies much higher than $1/(2\pi\tau)$, ε_S is the dielectric constant at very low frequency, and τ is the relaxation time. The relative complex permittivity ε is described by $\varepsilon = \varepsilon' - j\varepsilon''$. From equation 3, the dielectric constant and loss factor can be written as:

$$\varepsilon' = \varepsilon_\infty + (\varepsilon_S - \varepsilon_\infty)/(1 + \omega^2\tau^2) \qquad (4)$$

$$\varepsilon'' = \varepsilon_\infty + (\varepsilon_S - \varepsilon_\infty)/(1 + j\omega^2\tau^2) \qquad (5)$$

The conductivity of a dielectric with a single-time relaxation can be expressed with equations 2 and 5:

$$\sigma = \sigma_S + (\varepsilon_S - \varepsilon_\infty)\, \omega^2\tau\varepsilon_0/(1 + \omega^2\tau^2) \qquad (6)$$

At low frequency ε_∞ and σ_S are very small, then the relation between conductivity and dielectric constant can be described as:

$$\sigma/\, \omega\varepsilon_0\varepsilon' \sim \varepsilon''/\varepsilon' \sim \omega\tau \qquad (7)$$

$$C/G \sim \omega\varepsilon'/\varepsilon'' \sim 1/\tau \qquad (8)$$

where C and G are the capacitance and conductance of a material, respectively.

References

1 W. Kuang and S. O. Nelson, *Trans. ASAE*, 1998, **41**, 173.

2 K. K. Chi, *Dielectric Phenomena in Solids*, Elsevier Academic Press, London, UK 2004, p. 92-93.

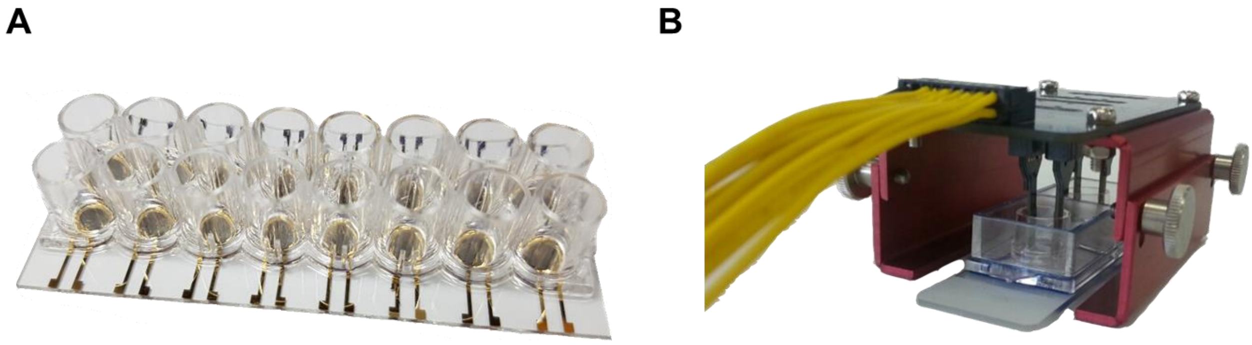

Fig. S1. Photos of the 2D and 3D impedance sensors. Photos of A) the 2D impedance cell sensor array consisting of 16 sensors and B) the 3D impedance cell sensor.

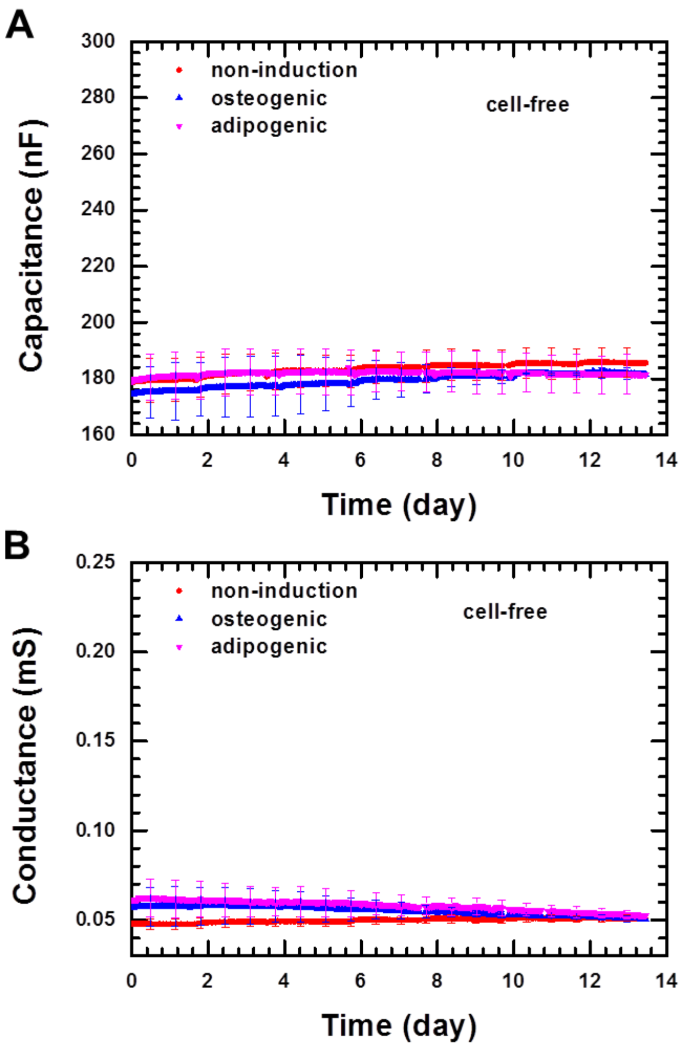

Fig. S2. Real-time capacitance and conductance for different types of cell-free medium in the 2D cell culture systems. A) Capacitance and B) conductance measured at f = 0.5 kHz over times without cells in the non-induction, osteogenic, and adipocyte induction media. Data are shown as means ± standard deviations (n = 3).

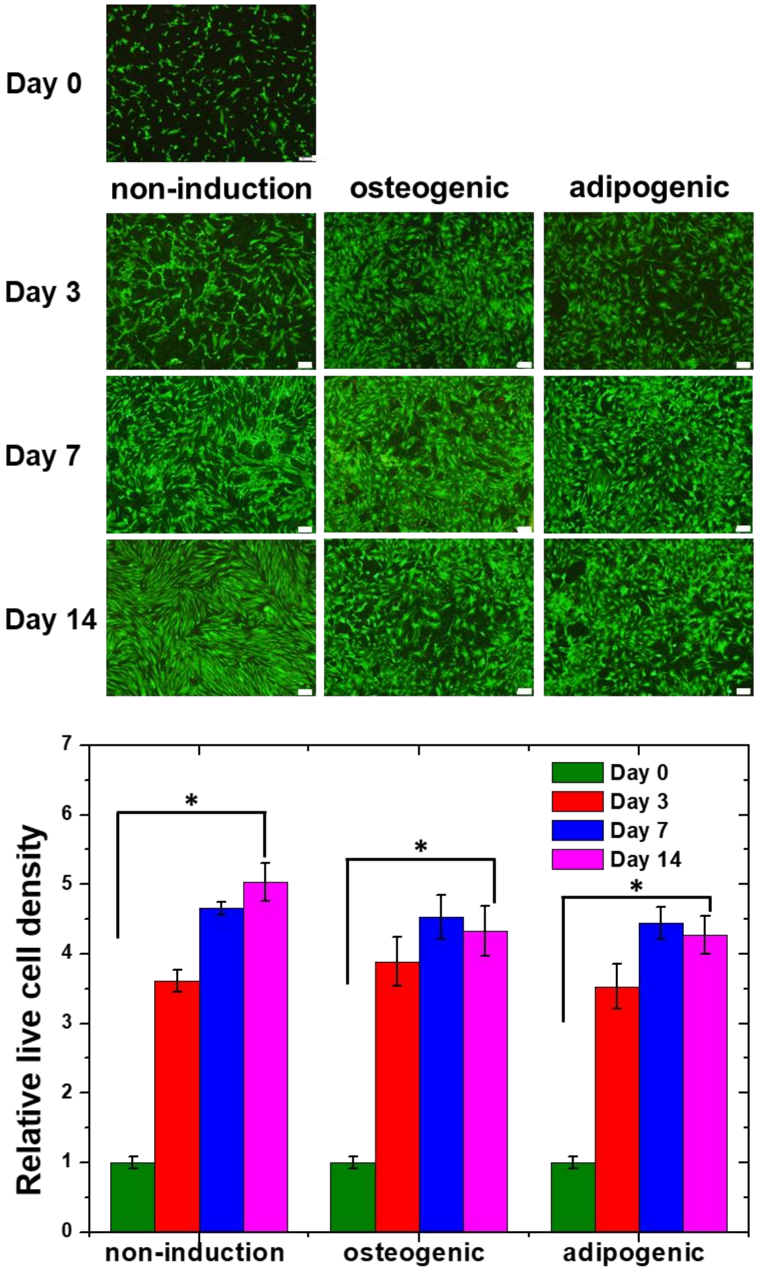

Fig. S3. Analysis of cell density of hMSCs in 2D cell culture systems. Time-lapse fluorescence images of hMSCs stained with ethidium homodimer-1/calcein AM after 2D cell culture in non-induction, osteogenic induction, and adipogenic induction media for different times. The right plot shows relative live cell density of hMSCs at different times following hMSC culture in 2D cell culture systems containing non-induction, osteogenic induction, and adipogenic induction media. Data are shown as the means ± standard deviations (n = 5). Scale bar, 100 μm. *$P < 0.001$

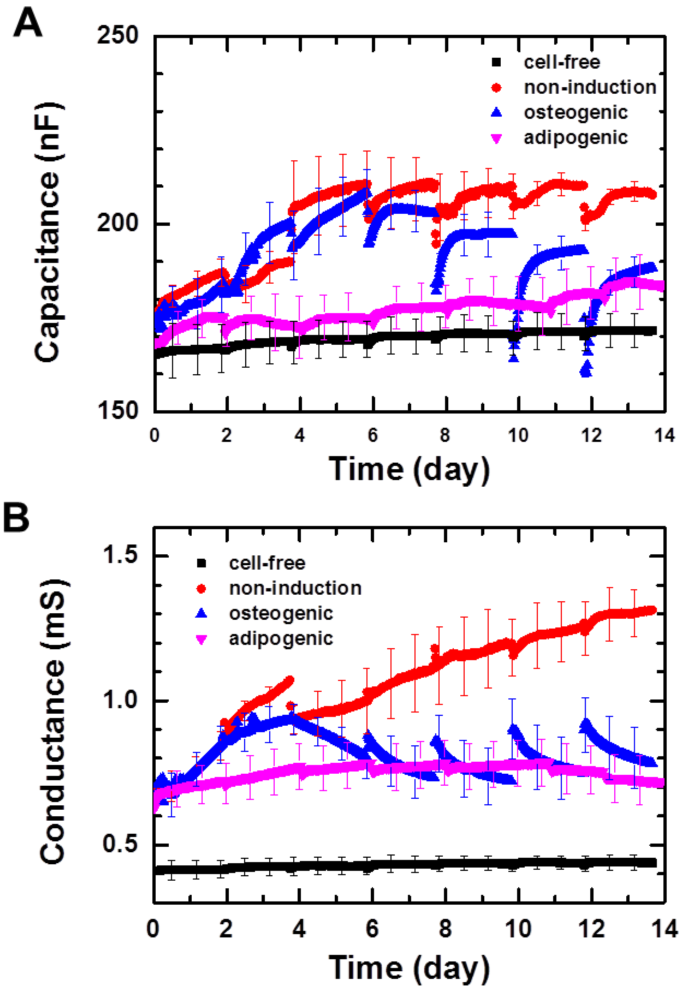

Fig. S4. Real-time capacitance and conductance of the 2D impedance cell sensor at f = 2 kHz. A) Real-time capacitance measured with the 2D impedance cell sensor. B) Real-time conductance measured with the 2D impedance cell sensor. Data are shown as the means ± standard deviations (n = 3).

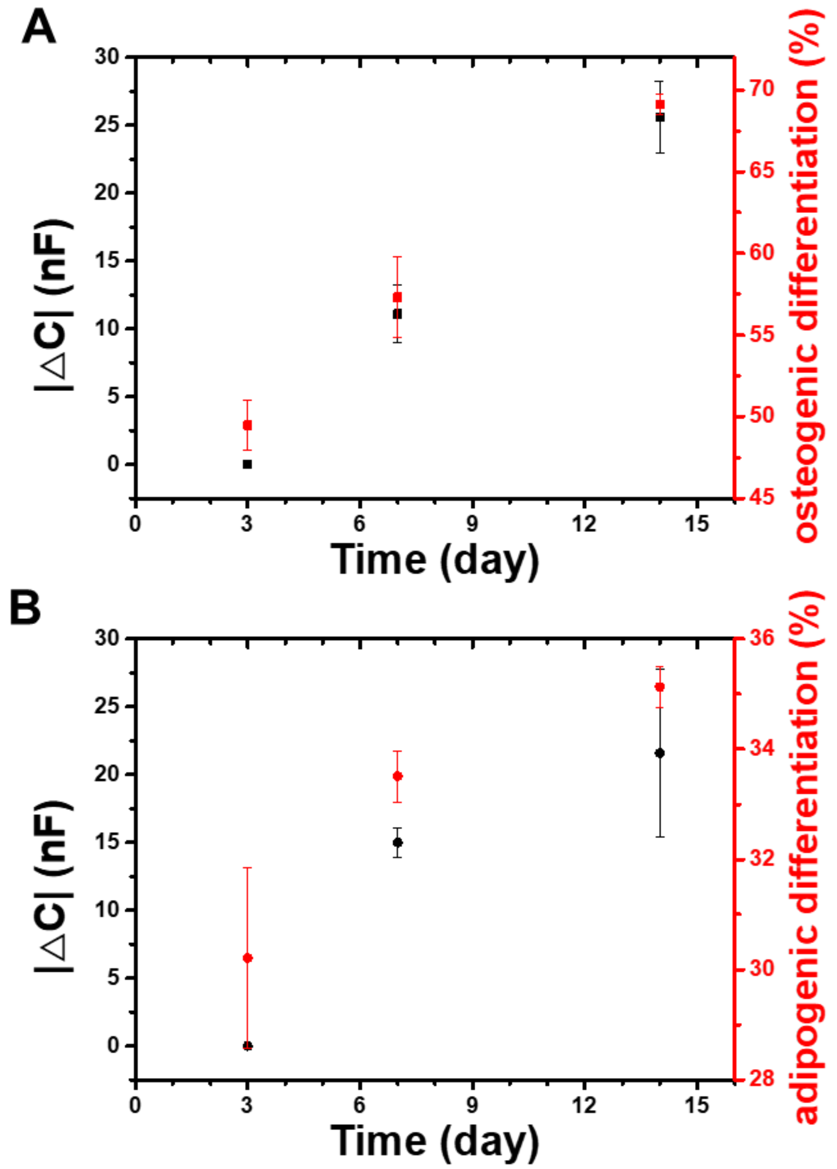

Fig. S5. Comparison between the capacitance change and the percentage of differentiated cells for 2D cell culture. The percentage of differentiated cells were estimated from time-lapse stained images and the capacitance change was calculated by subtracting the capacitance value at day 3 because differentiation appeared to start from day 3. Data are shown as means ± standard deviations (*n* = 3).

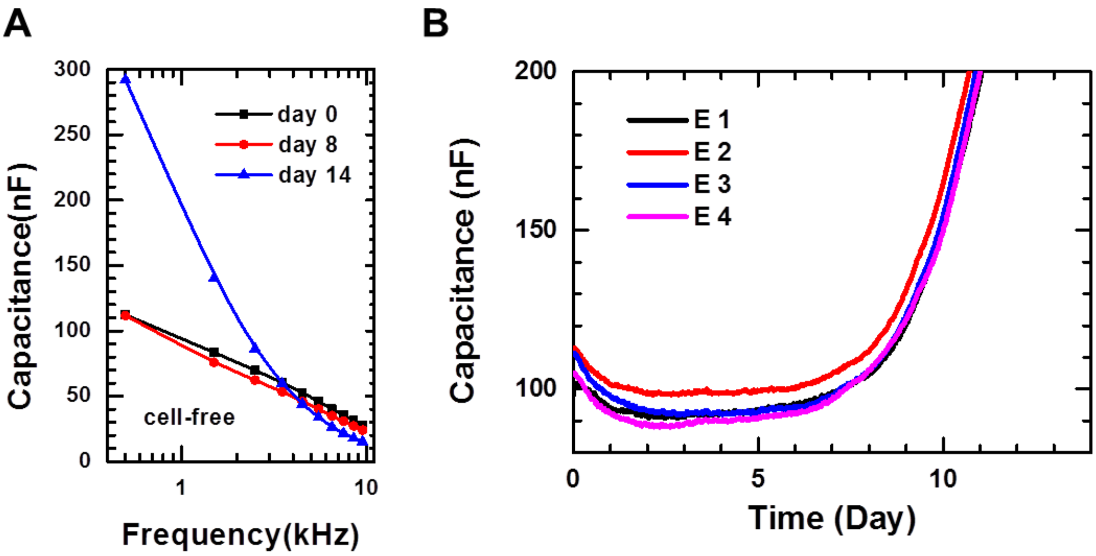

Fig. S6. Characterization of the capacitance of cell-free medium in the 3D cell culture system. A) Frequency dependence of the capacitance measured on days 0, 8, and 14 for the 3D impedance cell sensor with cell-free medium as a negative control. B) Time dependence of the capacitance as measured using four electrodes, E1, E2, E3 and E4, for cell-free medium at f = 0.5 kHz.

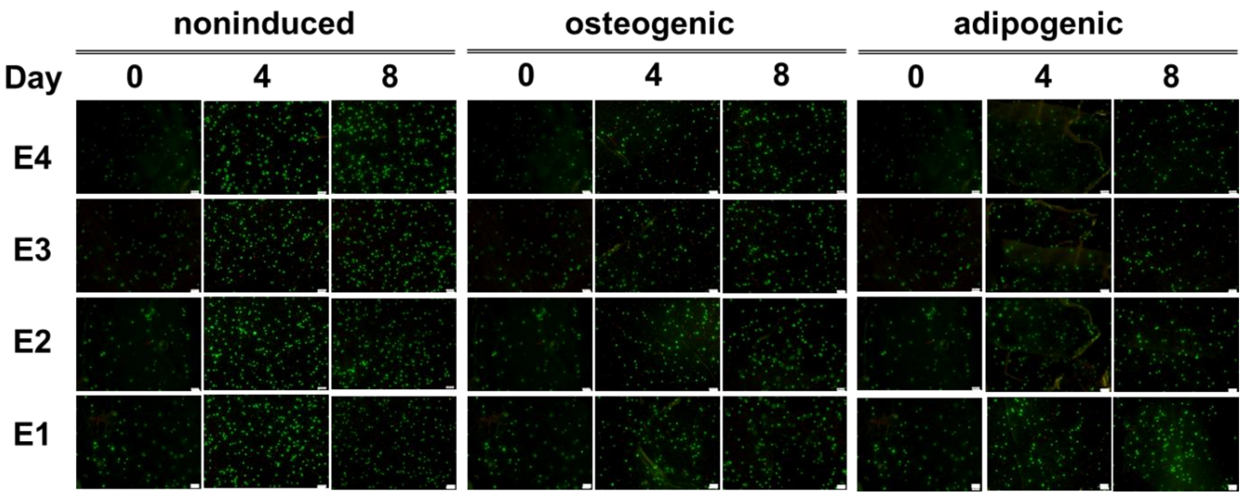

Fig. S7. Image of cell density of hMSCs in the 3D cell culture systems. Time-lapse fluorescence images of hMSCs encapsulated in hydrogel that was sliced horizontally and stained with ethidium homodimer-1/calcein AM after 3D cell culture in non-induction, osteogenic induction, and adipogenic induction media on the indicated days.

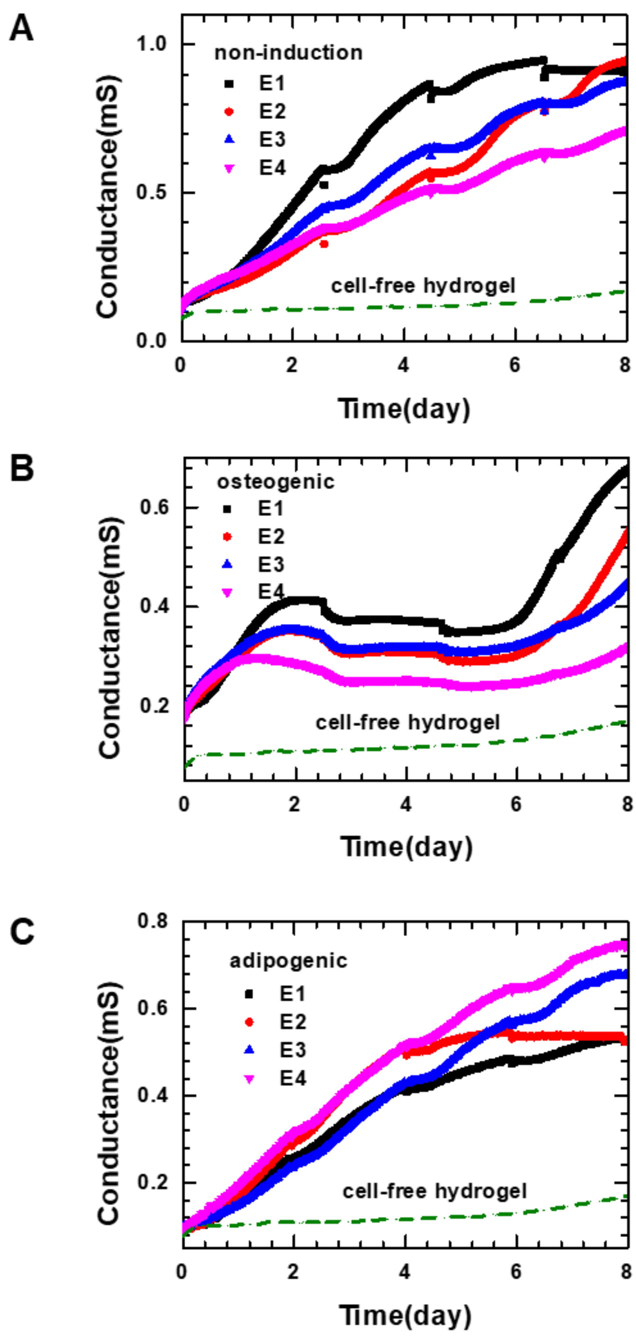

Fig. S8. Real-time monitoring of hMSC conductance in the 3D cell culture system at *f* = 0.5 kHz. The conductance of hMSC cell cultures measured over time using four electrodes, E1, E2, E3, and E4. The hMSCs encapsulated in hydrogel were cultured in the 3D cell culture system with A) non-induction, B) osteogenic induction, and C) adipogenic induction media. The dotted green curves show the conductance measured for the cell-free hydrogel (negative control).

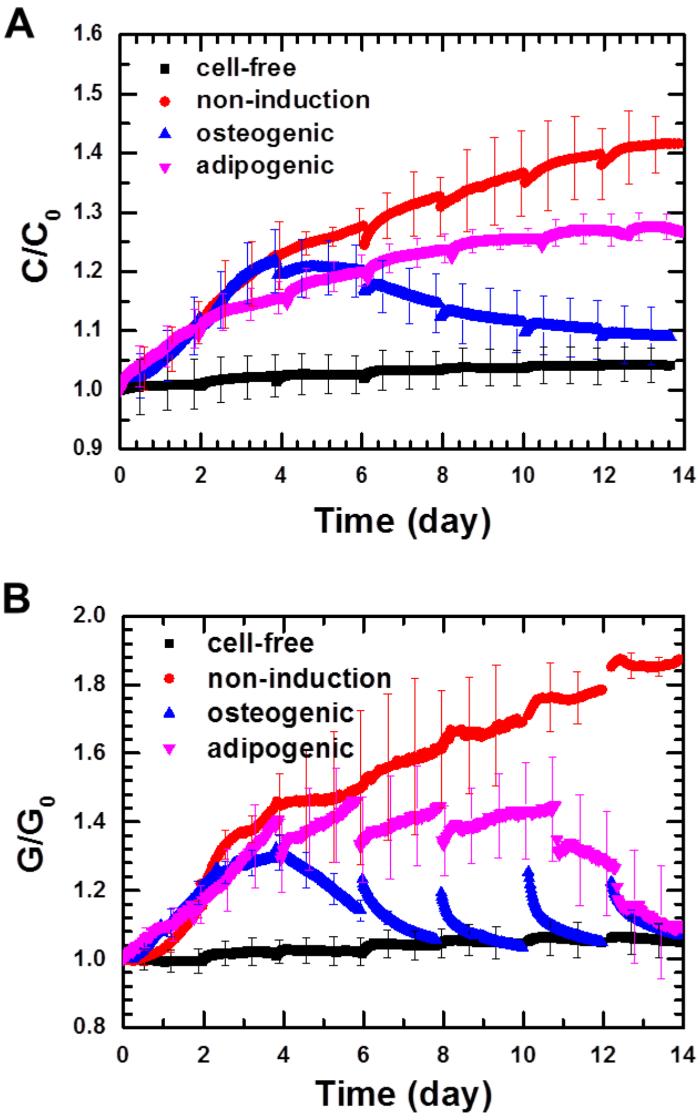

Fig. S9. Normalized real-time capacitance and conductance of the hMSCs in the 2D cell culture system. A) Real-time capacitance normalized by the initial values in the 2D cell culture system. B) Real-time conductance normalized by the initial values in the 2D cell culture system. Data are shown as the means ± standard deviations (n = 3).

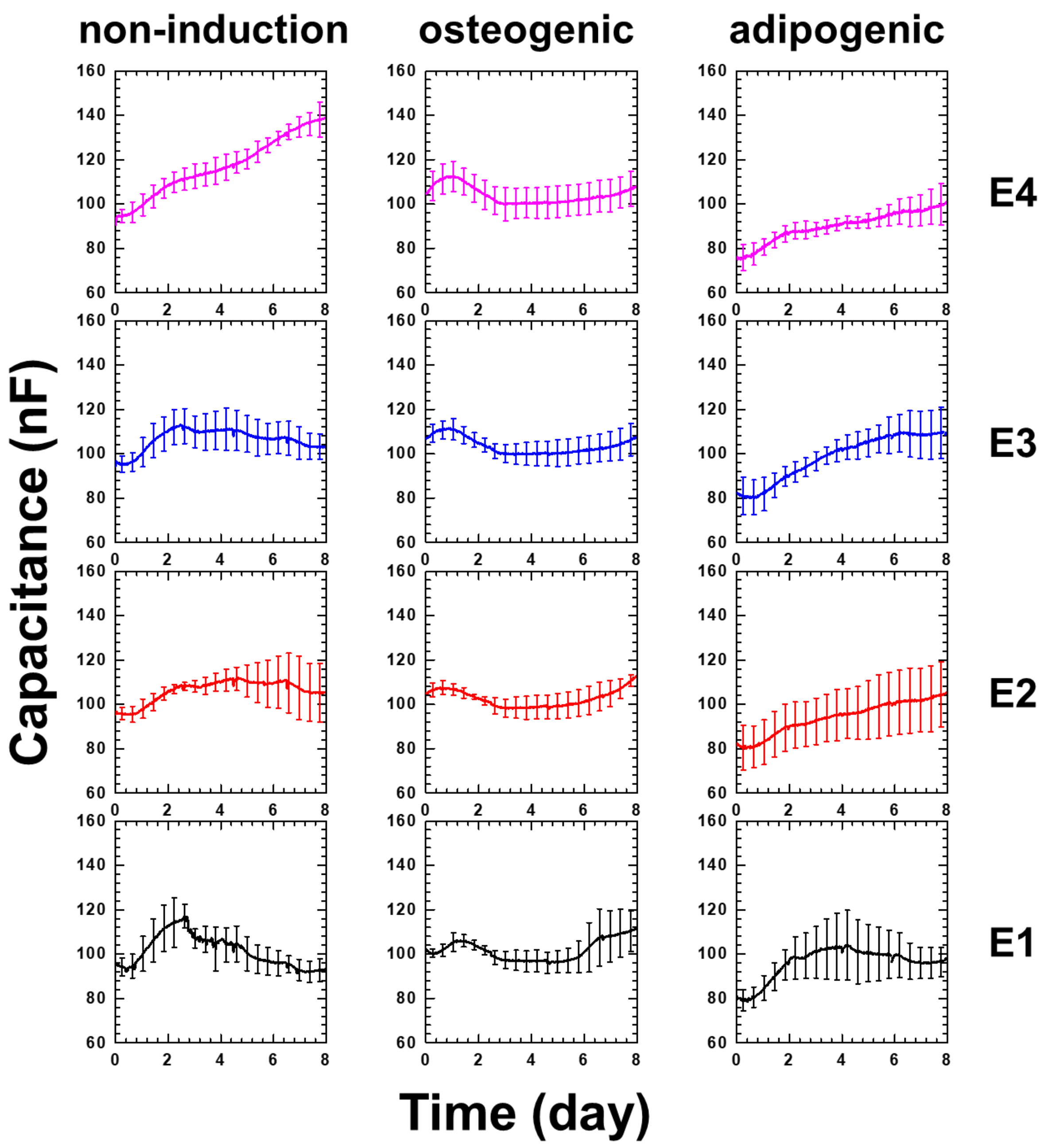

Fig. S10. Real-time capacitance of the 3D impedance cell sensor at *f* = 0.5 kHz. The real-time capacitance was measured with four electrodes in the 3D cell culture system. The data indicate the capacitance of each electrode in non-induction, osteogenic induction, and adipogenic induction media. Data are shown as the means ± standard deviations ($n = 3$).

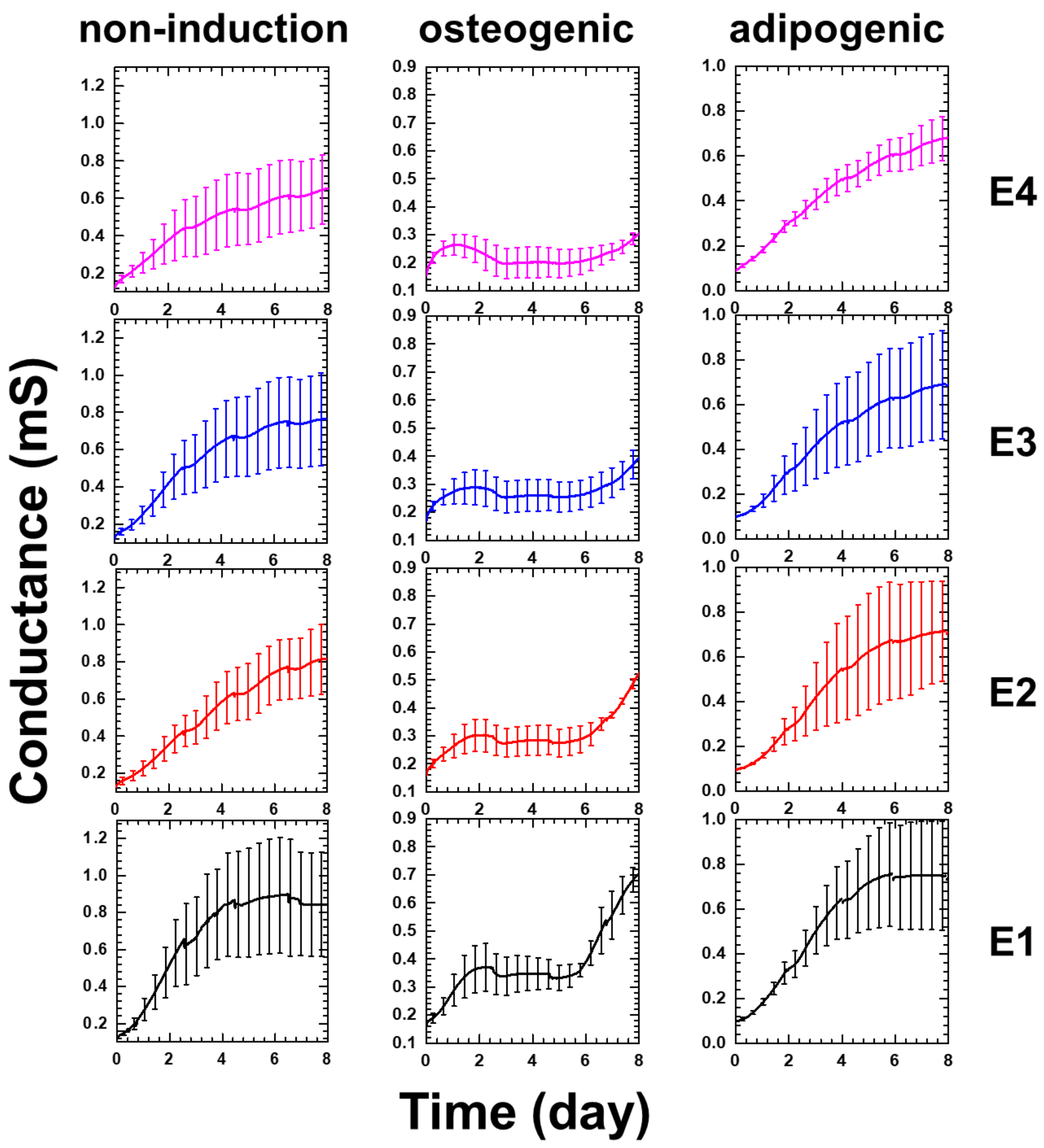

Fig. S11. Real-time conductance of the 3D impedance cell sensor at f = 0.5 kHz. The real-time conductance measured with four electrodes in the 3D cell culture system. The data indicate the conductance of each electrode in non-induction, osteogenic induction, and adipogenic induction media. Data are shown as the means ± standard deviations (n = 3).
